# Crowd-sourced cognitive mapping: A new way of displaying people’s cognitive perception of urban space

**DOI:** 10.1371/journal.pone.0218590

**Published:** 2019-06-20

**Authors:** Kee Moon Jang, Youngchul Kim

**Affiliations:** 1 KAIST Urban Design Lab, Department of Civil and Environmental Engineering, Korea Advanced Institute of Science and Technology, Daejeon, Republic of Korea; 2 KAIST Urban Design Lab, Department of Civil and Environmental Engineering, Korea Advanced Institute of Science and Technology, Daejeon, Republic of Korea; University of Turin, ITALY

## Abstract

By utilizing cognitive mapping and leveraging georeferenced text data, this paper aims to suggest a new visualization method that combines the advantages of both conventional and state-of-the-art research techniques to depict the collective identity of place in a single image. The study addressed two research questions: (1) Can crowd-sourced text data be utilized in representing place identity? (2) Can collective place identity be expressed in the form of a cognitive map? By confirming that text data gathered from social media effectively demonstrate people’s behaviors and perceptions related to places, we propose a novel method to create a visual representation of urban identity–a “crowd-sourced cognitive map”. In particular, to improve the conventional cognitive mapping method to depict the collective identity of a city, we draw cognitive maps of Bundang and Ilsan developed in the 1990s, as well as Songdo and Dongtan developed in the 2000s, just outside of the administrative boundaries of Seoul in Korea, through a computational method based on crowd-sourced opinions collected from social media. We open the possibility for the use of social media text data to capture the identity of cities and suggest a graphical image through which people without prior information could also easily apprehend the overall image of a city. The work in this paper is expected to provide a methodological technique for appropriate decision-making and the evaluation of urban identity to shape a more unique and imageable city.

## Introduction

Since its first appearance in the pioneering work of Proshansky [1], “place identity” has been redefined successively in conjunction with different contexts. He coined the term to describe “the substructure of a person’s self-identity with relation to the physical environment” and clarified the meaning as “clusters of positively and negatively valenced cognitions of physical settings” [2]. The term derives from the field of environmental psychology yet is currently regarded as a flexible idea that is often used interchangeably with different concepts that emphasize the interaction between people and their environment–namely, sense of place, imageability, placeness, place attachment, and place distinctiveness. Unlike the early emphasis on the physical settings of a place, place identity has more recently been conceived of as a gradient, “gestalt-like” concept consisting of multiple dimensions that include physical distinctiveness, social imageability, emotional attachment, and satisfaction [3]. This framework is a revision of the three basic elements of place identity proposed by Relph [4]: physical settings, activities, and meaning.

Discourse on place identity has remained a vital topic in urban studies in terms of the economic and social benefits it brings to the sustainable development of cities [5–7]. Unfortunately, cities are losing their individual identity amidst a worldwide trend of standardization in urban morphology resulting from economic and cultural globalization [8]. Described as “placelessness”, such a loss of identity was foreseen a few decades ago by Edward Relph [4]; however, little has been done to address it. In this context, the pursuit of place identity has become more important in modern mass-produced landscapes for interaction among people, as well as interaction between people and the surrounding environment [3].

Several research techniques have been used to assess place identity. Conventional qualitative techniques that capture people’s perceptions of place include survey, interview, participant observation, and cognitive maps but are prone to subjectivity, leaving concerns as to the reliability of results. Meanwhile, a new paradigm has emerged methodology-wise that has been triggered by the extensive use of sensor networks and wireless devices. In this new paradigm, humans can act as sociocultural sensors for the crowd-sourcing of urban form and function [9,10]. In recent years, many researchers have attempted to make use of such technological shifts in order to better understand the collective identity of place and the spatial perception of people [11–15].

By borrowing cognitive mapping and leveraging georeferenced text data, this paper aims to suggest a new visualization method that combines the advantages of both conventional and state-of-the-art research techniques to depict the collective identity of place in a single image. In this endeavor, the study addresses two research questions: (1) Can crowd-sourced text data be utilized in representing place identity? (2) Can collective place identity be expressed in the form of a cognitive map?

## Quantification approaches to place identity

How to quantify the subjective aspects of the urban environment, including place identity, has been studied across disciplines, including urban planning and design, geography, tourism, and environmental psychology. In the days when collecting data on a massive scale was a near impossibility, qualitative approaches, such as surveying [16–18], interviewing [19–21], and cognitive mapping [22,23], were key sources from which to obtain information about how people perceive their cities. One of the prominent examples of applying mapping exercises was conducted by Appleyard [24] to represent the relationship between the physical (traffic level) and nonphysical (community livability and social connections) attributes of the city. This work was taken over by his son, Appleyard [23], who explored the effect of traffic conditions on children’s perceptions toward the environment via a cognitive mapping approach. However, such qualitative approaches inevitably face the problem of subjectivity, as they barely cover a small portion of the related population.

In this sense, approaches in different directions were made to effectively assess place identity, by either visualizing qualitative data, or using different types of data. Sepe [25] used a mixed approach, termed “The PlaceMaker Method”, to construct a map that displays elements perceived by visitors to a place. Different types of data (e.g., sketches, traditional maps, surveys both verbal and graphical, questionnaires, etc.) were collected throughout the six phases of the overall protocol to suggest a novel cartographic image that identifies elements that construct place identity. Ruggeri [26] adopted a photovoice method to investigate place identity and attachment. The use of photovoice, where people are guided to take pictures of their community that highlight a certain research theme, was a method previously used in the fields of tourism and community design [27,28]. It was successful in discovering the identity of Mission Viejo, California [26], in conjunction with the operationalization of the concept “place identity”.

Today, in the era of big data, researchers are finding new ways to include excessive data in understanding the place-related perception of people. The crowd-sourcing of data has allowed the interpretation of collective perceptions to address the subjectivity problem in conventional research projects. Dubey et al. [12] collected the answers for pairwise comparisons of two different Google Street View images on perceptual attributes (safe, lively, boring, wealthy, depressing, and beautiful) to obtain and predict the “streetscore” of the site shown in the image. Liu et al. [15] confirmed the five elements of city image illustrated by Lynch [22] by interpreting the distribution of geo-tagged photos from Flickr and Panoramio and developed a scene attribute classifier to discover the “C-IMAGE,” the perceived image of the city, through a computational method. Cranshaw et al. [11] applied clustering models to the distribution of check-in venues of Foursquare users to define the dynamic boundaries of a city, “Livehoods,” which reflect the social flows of people in real life that do not correspond with municipal boundaries. In addition, researchers have used online tools to draw the mental map of neighborhoods [29] and used georeferenced picture tags to map all sort of aspects of the urban environment, including activities [30], ambiance [31], and senses [32,33]. The potential of other data types, such as taxi trajectories [34] and mobile phone usage records [13], have been largely examined as well.

## Methods

The widespread use of various photosharing websites and location-based services has paved the way for the application of data in place-related research projects. In particular, check-in locations and photo content are the two most commonly used types of data in measuring and quantifying people’s spatial perceptions. However, discerning the limitations of previous approaches to quantify and map place identity, this research is designed in two parts to address the drawbacks in terms of data type and expression method: (1) investigating the potential of text data in representing place identity, and (2) providing a new visualization that displays place identity in an intuitive picture.

Prior to starting this research, the three-component framework described by Relph [4] was adopted to clarify the scope of place identity. This framework is used to simplify the classification of textual data into each element for straightforward translation between the data and the theoretical concept. According to his definition, the components that form a persistent place identity are the place’s physical setting, activities, and events that occur at the place, and individual or group meanings aroused by their place-related experiences [4,35].

Although Instagram is the largest photosharing platform, the deprecation of its API endpoints has made Flickr, Foursquare, Panoramio, and Google Street View the major sources for spatial contents in recent studies. However, we select Instagram as the data source in this study, as it outnumbers its counterparts in the number of users within Korea. Instead of calling API endpoints directly from the platform, which is currently unavailable, we use an advanced Instagram search engine, Picodash (www.picodash.com). Picodash is a web-based paid service that complies with the Instagram Platform policy to allow users to export Instagram data of followers, hashtag posts, location posts, comments, etc. Furthermore, its terms of service clarify that data is provided for research and analysis purposes unless it is reposted or used by unauthorized means. Metadata exported from Picodash comprises 20 columns of information that include the creation date, caption text, location value (latitude/longitude of upload location), location name, and so on. Data were searched under specific location terms. More specifically, metadata of Instagram photos that contain “#location_name” was exported to form the primary dataset, from which we extract additional hashtags in caption text of the resulting photos for further analysis. These hashtags are collected on the assumption that they directly reflect users’ opinions on the location used as the search keyword and exist in a single word unit, which enables simple processing.

**Fig 1:**
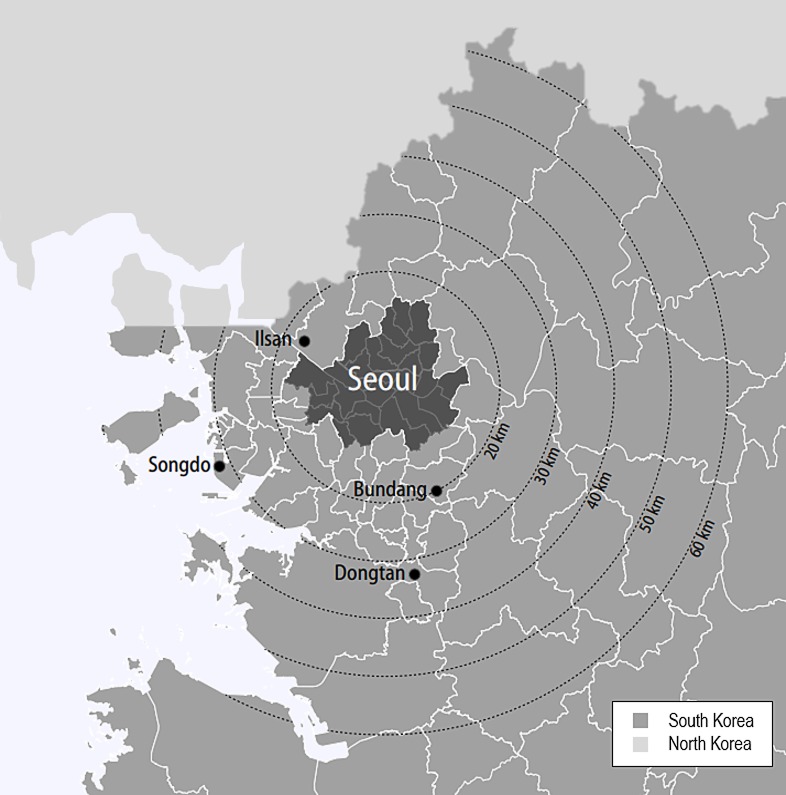
Map of the seoul metropolitan area and locations of the four case cities.

In this study, analyses are conducted on four case cities located in the Seoul metropolitan area: Bundang, Dongtan, Ilsan, and Songdo. The locations of the four sites are shown in Fig 1. They were constructed as part of Korea’s new town development plan throughout the 1990s and 2000s, whose purpose was to disperse population and lower housing prices in the nation’s capital [36]. The last few decades, however, have transformed them into different places that not only possess but are also perceived as cities with individual characteristics. Bundang is frequently referred to as “a city beneath heaven”, which implies its livable urban environment. Currently it is transforming into an innovative city with the development of Pangyo district, a techno-valley that is attracting and supporting various R&D centers and start-ups. Dongtan is a residential town located on the outskirt of the central region, successfully carrying out the purpose for which the new town was intended. Songdo aims to gain the title of Asia’s ‘global economic hub’ by attracting foreign capital through promoting its state-of-the-art smart city scheme and nearby Incheon airport. Ilsan, one of the first-generation new towns, is now functioning as an autonomous suburban community with its own administrative and cultural functions. Such similarities in their development backgrounds and differences in their city image and functions today will be examined throughout the study.

Based on the premise that texts deliver user perception more directly than other forms of social media data (photos, geo-location, etc.), we construct a dataset using hashtags from Instagram photos, from which identity-related meanings are extracted to validate the potential of texts in discovering place identity. Furthermore, we extend the investigation into a visual representation of the collective identity of a city, namely, “urban identity,” at a single view. For this purpose, we borrow the form of a cognitive map, which has been widely used to portray people’s perception of the environment. Symbols and written text in cognitive maps indicate locations and explain detailed information, respectively, and are substituted by visual expressions of identity-related meanings extracted from hashtags. Significant locations, which contribute to the place identity as a “physical setting” element, are discovered through analysis. The same process of data collection and class assignment is conducted for those locations as well, to extract “activity-” and “meaning-”related perceptions expressed within the cognitive map.

## Discovering identity-related meanings from texts

To answer the research questions of this study, we begin by revealing the possibility of texts in quantifying urban identity. Data were searched and collected under the names of the chosen new towns, Bundang, Ilsan, Songdo, and Dongtan, from the Picodash website. To ensure a sufficient amount of data for cities in Korea, data search and collection were conducted in Korean, then translated into English for the processing and visualization steps. For Bundang, a total of 288,641 datapoints uploaded between 08/25/2016 and 06/01/2017 were obtained to construct the primary dataset; for Ilsan, 971,446 datapoints between 05/25/2016 and 08/05/2017 were collected; for Songdo, 187,044 datapoints between 03/31/2015 and 08/05/2017 were collected; and for Dongtan, 338,637 datapoints between 03/31/2015 and 07/25/2017 were collected. Among the twenty different columns in the exported CSV file, applicable data come from the column of “caption_text”, which consists of freely written text and a sequence of hashtags. However, this study solely utilizes the hashtag sequence, as it facilitates semantic extraction, whereas its untagged counterpart requires a complicated text mining process that is unnecessary for the purpose of this study. Collected hashtags are keywords that appear with the location term, intentionally added by users to directly reflect their opinions. Stopword removal follows to get rid of irrelevant Instagram-related keywords (e.g., insta, stagram, follow, like, ootd, etc.). Lastly, by counting repeated keywords, a list of hashtag keywords and frequency was constructed. For further analysis, we correlate this dataset with the three elements of place identity suggested by Relph [4] by assigning a place meaning to each keyword. For example, if a keyword indicates a “physical setting,” or simply a location, we assign a class value of L. Likewise, a keyword that explains an “activity” or “meaning” is assigned A or M respectively. Those keywords that do not fall into any one of the three elements are given a class value E, which stands for “else.”

**Fig 2:**
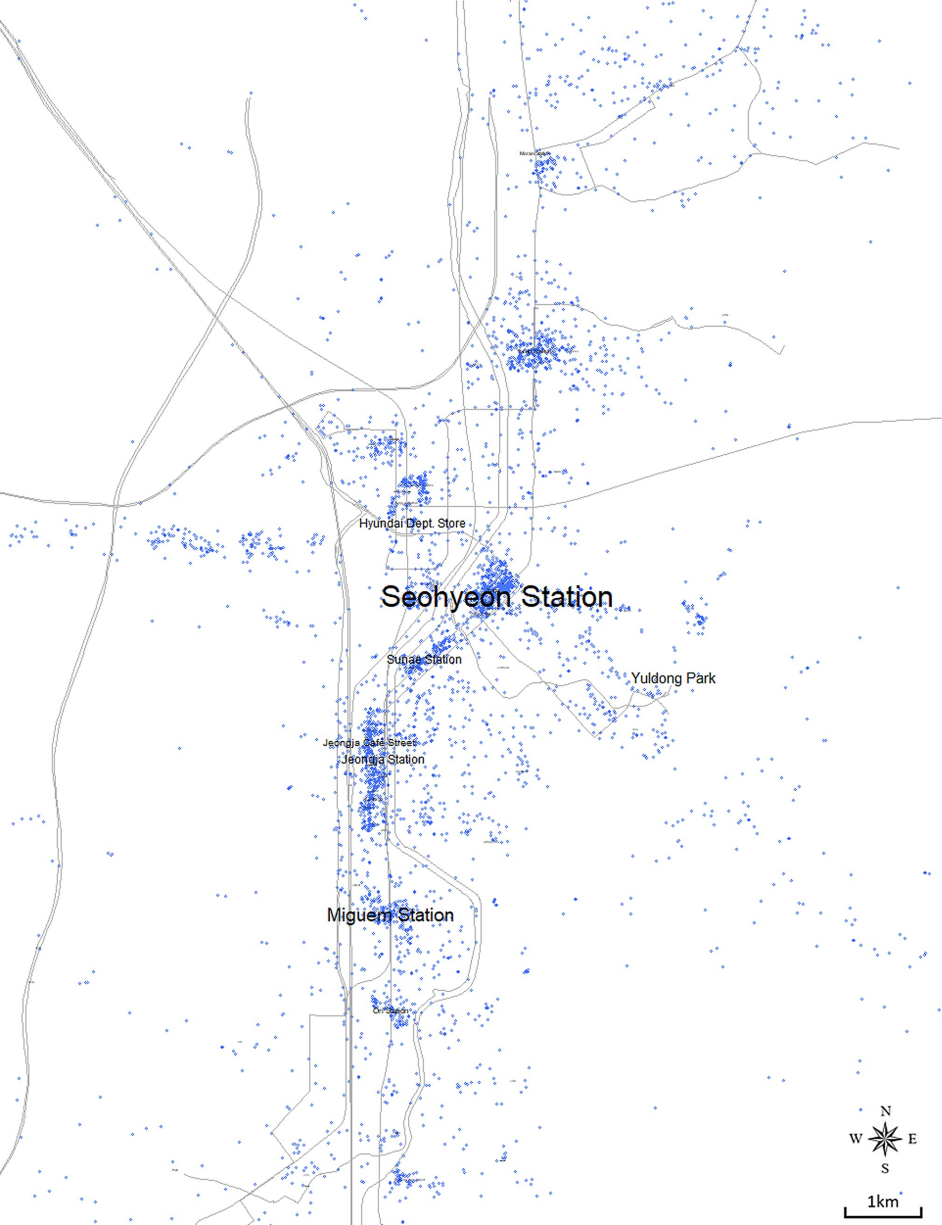
Instagram check-in points overlaid with the distribution of “location” keywords in Bundang.

We tested the potential of text data by overlaying the distribution map of check-in points with that of “Location” keywords, which we refer to as a “location-text distribution map” in this part of the study. Within the metadata, a total of 92,236 items of Bundang, 260,375 items of Ilsan, 187,044 items of Songdo, and 89,413 items of Dongtan possessed check-in locations. For example, Fig 2 shows the overlay image of check-in points and the distribution of “Location” keywords in Bundang. Detecting the formation of point clusters has been a widely used method in previous urban research projects to determine spatially significant locations within a city. Such clusters were interpreted as urban spaces that contribute to the formation of place identity; thus, nonphysical characteristics, such as function, role, or significance of these clusters, were analyzed. However, considering the behavior pattern of photosharing users, we are empirically aware of cases in which the check-in location is irrelevant to the uploaded content. Fig 2 identifies this inconsistency, where we scattered L-class keywords on the actual locations that tags signify in a size based on their relative frequency. Texts in the figure come from hashtags added in connection to the city name “Bundang”, which in turn we can understand as cognitive information directly related to the city. However, a few texts with a notably large size failed to overlap with point clusters; conversely, a few highly dense clusters failed to coincide with a legible text. In Fig 2, Yuldong Park, one of the second largest texts in the figure, appears on a nearly empty space on the right side of the figure. Similarly, Hyundai Department Store does not overlap with any cluster as well, existing only near a cluster at Pangyo subway station. While check-in point clusters are regarded as locations contributive to city identity, the mismatch between the two data reveals the limitations of the traditional point-based approach and confirms the potential of text data in discovering significant locations. This inconsistent pattern may derive from either the spatial characteristic as an outdoor area, or the behavior pattern of people visiting the location. For example, an unstable Wi-Fi connection or the desire to enjoy the atmosphere in outdoor spaces prevents people from using wireless devices and sharing photos in real-time. Instead, people will return to their everyday spaces and satisfactorily upload a photo using “#Bundang #YuldongPark”. In this sense, we indicate that a point-based map cannot fully identify spaces that are perceived as spatially significant, but rather text data and their frequency combined with spatial information could fill the gap. Dunkel’s study [37] supports this finding by highlighting the effectiveness of tags, which are consciously added to social media content, in capturing landscape perception for future urban planning. This analysis proves that text data from photosharing social media, more precisely hashtags, can compensate for the shortcomings of conventional point-based approaches.

## Crowd-sourced cognitive map

By confirming that text data gathered from social media effectively demonstrate people’s behaviors and perceptions related to a place, we extend the investigation by proposing a novel method to create a visual representation of urban identity–a “crowd-sourced cognitive map”. The term mirrors one of our research goals to improve the conventional cognitive mapping method to depict the collective identity of a city. Therefore, we draw a cognitive map through a computational method based on crowd-sourced opinions collected from social media. In each case, i.e., Bundang, Ilsan, Songdo, and Dongtan, different hashtag keywords and their frequencies were extracted from the metadata of each case. We assign class values to each of the keywords and attempt to visualize the data in a cognitive map form that explains the identity of Bundang, Ilsan, Songdo, and Dongtan.

### Location, activity, and meaning of the four cases: *Bundang*, *Ilsan*, *Songdo*, *and Dongtan*

#### Physical setting

Unlike traditional maps that are primarily made for delivering the objective information of a region, a cognitive map contains subjective information of “where has what sort of meanings”. Accordingly, the cognitive map does not necessarily display all locations of the site; rather, selective locations of focus are displayed with descriptive explanations. Thus, to draw a cognitive map based on crowd-sourced data collected from social media, we must develop an idea of how to determine a number of locations that are collectively perceived as significant spaces. By suggesting the potential of text data in identifying the collective cognition of the city, this study seeks to conceptualize a standard to define the “physical setting” elements of place identity based on the overlay image in Fig 2. The inconsistent pattern between two data types–check-in points and hashtag keywords–produces a four-choice situation. Locations that feature a high volume of point data and a large size keyword are inevitably selected as significant spaces. After clarifying the limitation of point-based approaches, we reason that the frequency of textual data provides a better indicator than point density. Nevertheless, the point clusters that appear, which were interpreted as significant spaces in previous studies, still have meaningful implications and should not be ignored.

As a result, three criteria are adopted as follows: (1) High text frequency & High point density; (2) High text frequency & Low point density; and (3) Low text frequency & High point density. For example, according to the criteria, six locations *in toto* are selected as “location” elements that contribute to the identity of Bundang. Seohyeon station, Jeongja-dong Café Street, and Miguem station fall into the first category; in the same way, Yuldong Park and Hyundai Department Store fall into the second category and Yatap station into the third. These locations constitute the basis for creating a crowd-sourced cognitive map.

#### Activity & meaning

While Relph, in his book *Place and Placelessness*, specified the three components of place identity as physical setting, activity, and meaning, meaning is mentioned as a particularly elusive concept, whereas the other two components are easily recognizable [4]. He adds that this elusiveness derives from the complexity and variation that result from the reflective properties of meaning that mirror people’s intention and experience. In terms of place, function (or purpose), properties, and sentiment (or emotion) are the most likely subcategories of “meaning”, but fail to be clearly distinguished among themselves, or from the other two elements of place identity. Thus, to avoid ambiguity, activity and meaning keywords are displayed in the cognitive map without separation.

We extract semantics in activity and meaning keywords at two different scales. First, general semantic information is captured from the whole city. Second, the six locations are identified to contribute to the place identity of each case. For the finer scale analysis of major locations in Bundang, Ilsan, Songdo, and Dongtan, data were collected using each location as a search term. Furthermore, Table 1 lists keyword examples assigned to the classes of activity and meaning, which were categorized into twelve subcategories. This classification is an extension of previous studies which attempted to summarize different dimensions of place values [26,38,39], and aims to effectively separate activity and meaning keywords collected in this study.

**Table 1:** Subcategories of “activity” and “meaning” keywords, with specific examples for each category.

Activity/Meaning category	Color	
Culture/Art	hotpink3	Busking, Concert, Dept. store cultural center, Hiphop, Kidult, K-pop, Movie, Museum of Kid’s Books & Arts
Eating	cornsilk	Drinks, Beer, Brunch, Café, Chicken and Beer, Cocktail, Coffee, Dessert, Draft beer, Ice cream, Korean barbecue, Pub, Shake Shack, Soju, Starbucks, Sushi
Sports/Athletics	red	Ballet, CrossFit, Cycling, Golf, Health, Mixed martial arts, Pilates, Rehabilitation, Stretching, Weight training, Workout
Work	gray50	Company dinner, Company lecturing, Job interview
Outdoor/Nature	springgreen4	Bungee jumping, Drive, Lake, Merry-go-round, Outing, Picnic, Scenery, Terrace, Walk
Socializing	cyan	Birthday party, Blind date, Charity bazaar, Chat, Club activity, Dating, Party, Relationship
Sightseeing/Local	khaki3	AK plaza, Book theme park, Bundang subway line, Kakao Friends
Consumption/Refreshment	blue	Action figure, Cosmetics, Pop-up store, Present, Relaxation, Shopping, Vibe
Hobby	yellow	Karaoke, Photography, Pottery Studio
Family Activity	orange	Daughter, Family, Parenting, Pet dog, Son
Seasonal/Temporal	skyblue1	Autumn, Children's day, Christmas, Evening, Spring, Weekend
Emotion	darkslateblue	Love you

To visualize the activities and meanings that arise within the city, we adopt the graphical shape of concentric circles, where each circle represents a different activity or meaning. To provide a clear comparison among keywords and reduce visual clutter, the relative diameter of each circle is derived from the difference in the square root of the frequency of its representing keyword and the minimum value that appears in expression results. Here, we intend to express the pervasive impact of urban activities and meanings on how people perceive certain locations via the area of circular representation that is linear to frequency values. The color of a circle indicates the detailed classification listed in Table 1. Colors were selected from the list of colors available in R software. By plotting a multicentric map, we seek to substitute written texts in conventional cognitive maps via a visual method that descriptively explains the subjective information of multiple locations. Nearly all of the twenty most frequent keywords were used in plotting concentric circles; the remaining keywords were omitted for cleaner visualization.

**Fig 3:**
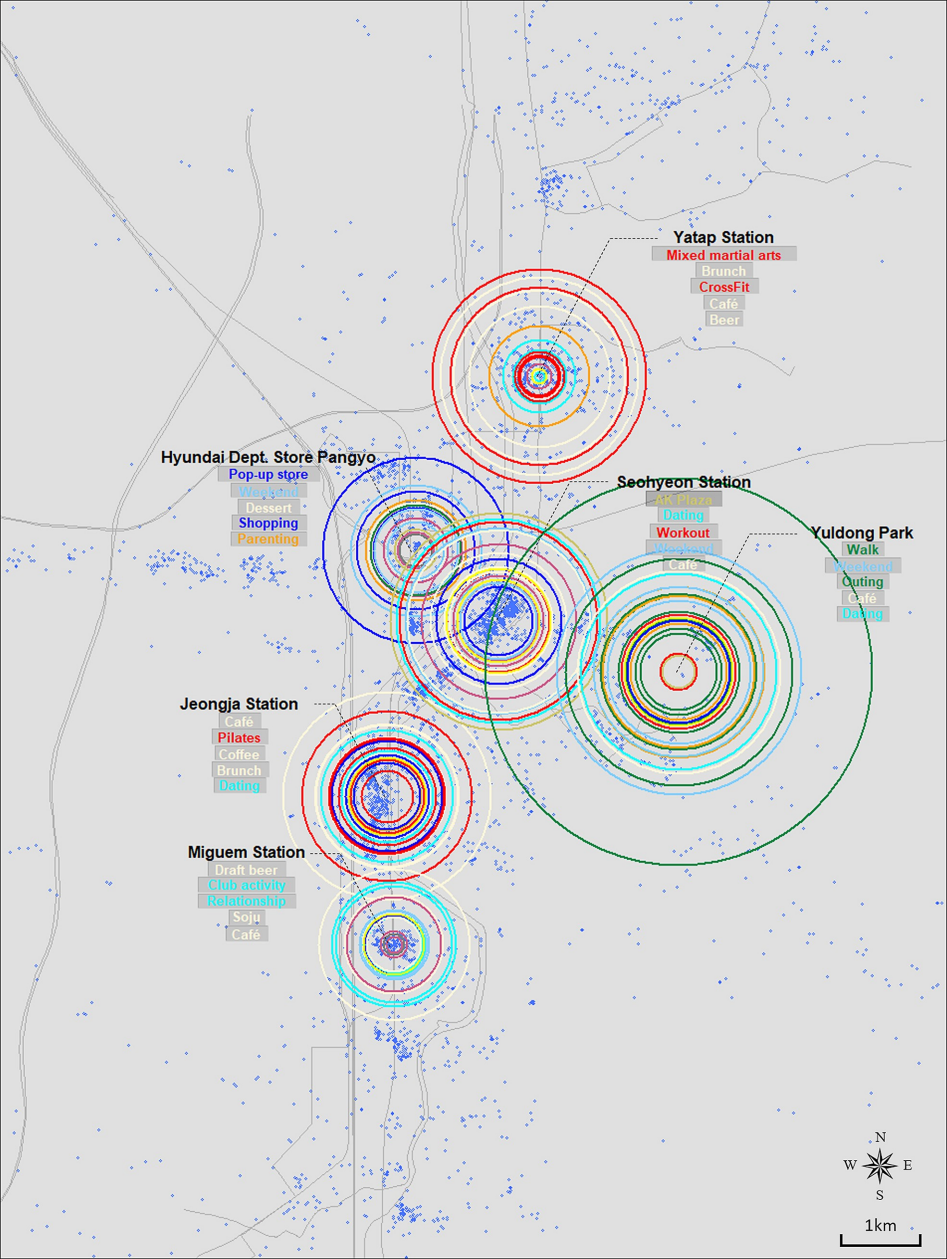
Crowd-sourced cognitive map of Bundang.

**Fig 4:**
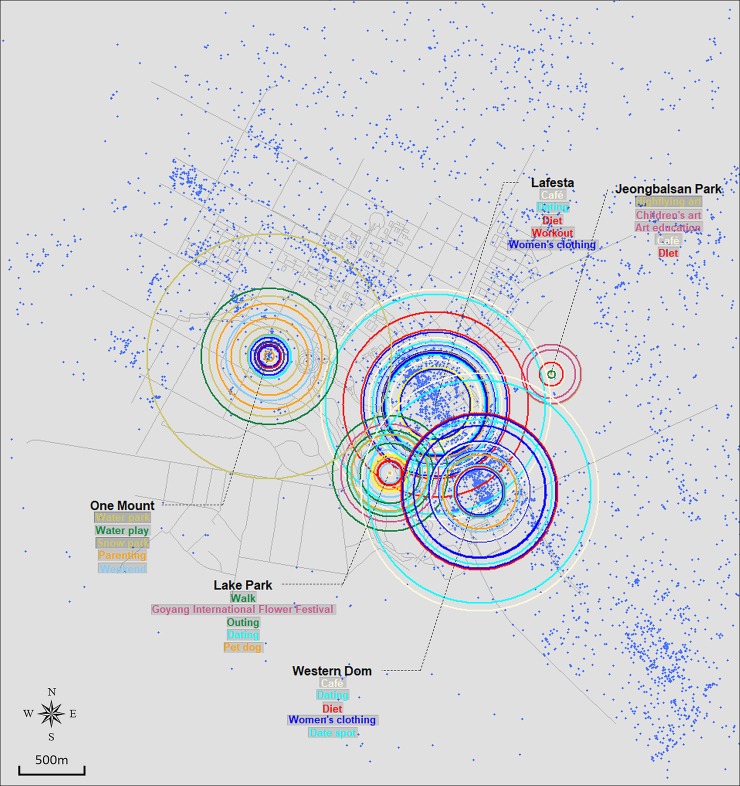
Crowd-sourced cognitive map of Ilsan.

**Fig 5:**
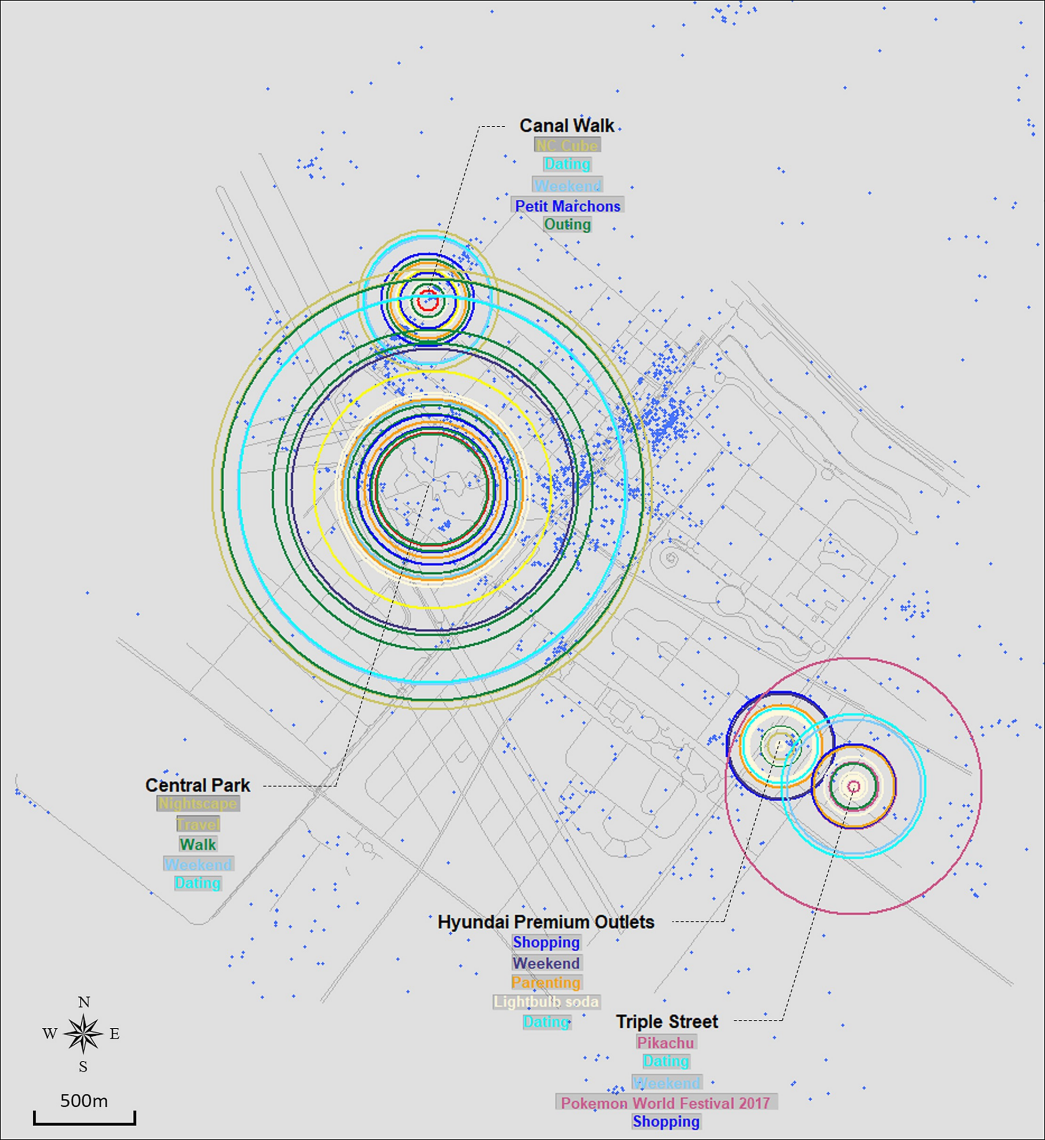
Crowd-sourced cognitive map of Songdo.

**Fig 6:**
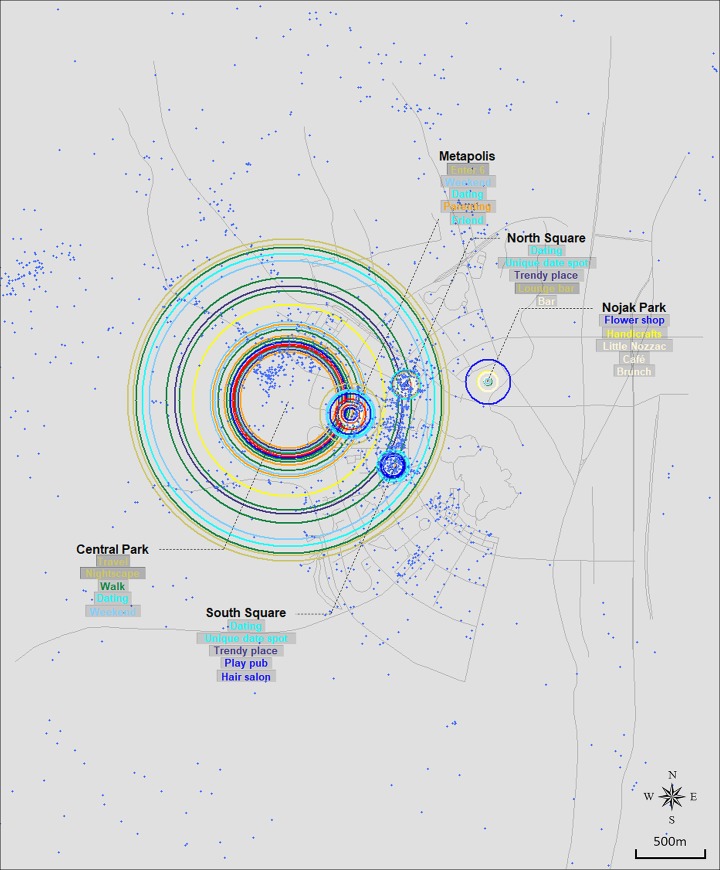
Crowd-sourced cognitive map of Dongtan.

Figs 3, 4, 5 and 6 show the resulting crowd-sourced cognitive maps of Bundang, Ilsan, Songdo, and Dongtan. From the overall image, differences among locations, such as size distribution and color composition of circles, are identified. The top five elements in terms of frequency are listed for each location. This method indicates that while several locations likewise participate in the formation of collective urban identity, they are perceived as separate places in terms of available activities, induced emotions, or visiting purposes.

### Details and discussions at the major locations in Bundang, Ilsan, Songdo, and Dongtan

Innumerable translations of the crowd-sourced cognitive map in the above context are possible. However, as each circle denotes a single keyword, the same colors do not necessarily represent the same activities or meanings. In this sense, we zoom into each location, one by one, and investigate the content in greater detail. For a clearer statement, the emphasis of this analysis is not on identifying the meaning of every keyword, but instead on ascertaining if there are any distinctive elements or meaningful relationships among keywords.

#### Bundang

The three subway stations, Yatap, Seohyeon, and Miguem stations, have the identical ostensible function of being a major transportation point of Bundang, but their color composition implies how people differently recognize each station in Fig 3. Miguem station mostly includes circles of cornsilk (eating) color, which implies that people recognize this area as a good place to dine. Red (sports/athletic activity) is notable at Yatap station, meaning that athletic activities are commonly engaged in near the area. On the other hand, Seohyeon station shows an even mix of color, evoking many different types of activities and meanings. In terms of size, Miguem station is smaller than the other two, which indicates its lesser importance to or lower level of awareness of the people mentioning Bundang. Similar interpretations are possible with the other locations. To point out some noticeable observations, green (outdoor/nature) is dominant in Yuldong Park, with skyblue (seasonal/temporal) as the secondary color. The combination of the two could be understood as people perceiving the park in relation to a certain time or season. Note the size of the outermost green circle that covers most of the city area; this shows how influential the park is to people when they think of Bundang. However, when zoomed out to view the entire image, we can observe the predominance of cornsilk (eating) color throughout the map. This likely results from the strong tendency of Instagram usage to upload food- or restaurant-related content.

#### Ilsan

The crowd-sourced cognitive map of Ilsan in Fig 4 consists of five sets of circles that are adjacent to one another. This configuration describes the spatial structure of the city as having a central district where most of the major facilities are aligned with Lake Park. Lafesta and Western Dom, two open-street shopping malls within walking distance, are located in the center of Ilsan. The size and color distribution of the two locations are nearly identical, proving that they are closely related to each other. One Mount also covers an area as large as the two shopping centers. Although it is a multipurpose building, it is best known for the waterpark and snowpark that are open year-round. Such unique features result in the formation of a khaki-colored circle (which denotes a local element) at the outermost layer. Lake Park and Jeongbalsan Park are expressed by comparatively small sets of circles. Although they are the major green infrastructure of the city, they do not grow large enough to be more popular than commercial areas. A relatively small size for green areas is a contrasting result to that observed in Bundang and Dongtan. The finding that cities present different size distributions among urban functions implies how cities have developed to shape distinctive place identities.

#### Songdo

In Songdo, five locations were selected as spatially significant locations. A point cluster observed in the middle of the map corresponds to Convensia Street which, however, was unavailable to be plotted due to insufficient data collected under the term “#Convensia Street”. Therefore, the remaining four were expressed with arrays of concentric rings comprising the crowd-sourced cognitive map of Songdo in Fig 5. Similar to Dongtan, Central Park covers the largest area, while the other commercial districts have relatively local-scale sets of circles. This size difference explains the profound impact of the park on the identity of the city. The three shopping centers–Canal Walk, Hyundai Premium Outlets, and Triple Street–opened their doors in recent years, resulting in a size that is thus far not considerable. However, owing to an international animation festival that took place in the most recently built shopping mall, “Triple Street”, its contents are visualized to a larger extent compared with the other two. This has implications regarding how a certain cultural event or other influential element could promote the creation of place identity at a rapid rate.

#### Dongtan

Although five significant locations were identified through the overlay analysis, Central Park overpowers other sites within the city. The size of its concentric circles is extraordinary, and green colors in the outermost circles imply its main function for sightseeing and outdoor activities. However, such colors are not evident in the case of Nojak Park. Instead, blue and cornsilk appear to be the main colors, which provide implications for the actual visiting purposes of people to the park. The second largest set of circles is centered on Metapolis with a comparatively even color distribution. This reflects the building’s usage as complex residential towers equipped with a variety of restaurants, shopping malls, movie theaters, etc. The other two, North Square and South Square, are pedestrian precincts, almost identical in their physical space and both surrounded by several commercial buildings. As such, keywords in these two locations mostly fall into categories of socializing, eating, or consumption. Nevertheless, slight differences can be seen from their contents, with North Square being more focused on eating, while South Square has more consumption activities.

### Implications for the identities of newly developed cities in Korea

While the contents of each case has been discussed in detail in the previous section, comparing the global form of maps carries significant implications as well. Although the four cities can be broadly classified as new towns in Korea, the former two and the latter differ in terms of when they were developed. Bundang and Ilsan are two of the five cities included in the first generation new town development plan in the 1990s. On the other hand, Dongtan’s and Songdo’s development followed in the 2000s and is ongoing. This difference is revealed through the size distribution of graphical elements in Figs 3, 4, 5 and 6. Figs 3 and 4, which are crowd-sourced cognitive maps of Bundang and Ilsan respectively, show that visuals representing each location’s place identity are displayed in a nearly even distribution in terms of their size. Such a pattern implies the completion of place identity formation in multiple locations in new towns now entering their fully developed stage. This can be translated as the building-up process of urban identity on a citywide scale discernible via the combination of place identities manifested in key locations within the city. In contrast, a clear distinction is observed between locations in Figs 5 and 6. In particular, Central Park stands out in both figures of Songdo and Dongtan, characterized by keywords mostly from the Sightseeing/Local and Outdoor/Nature categories. The prevalence of green open spaces over other functions in cities in their initial stage of development conforms to the history of new-town development in Korea, during which cities made slow progress in becoming self-contained with a sufficient urban infrastructure, but rather have developed into commuter towns. Hence, interpreting the crowd-sourced cognitive maps suggested in this study can provide indications of the current state of urban identity formation in cities.

## Conclusion

For its benefits to the sustainable development of cities, place identity has been emphasized in discussions regarding urban environment. To assess and measure the intangible concept that lies within human perception, previous studies have relied on qualitative approaches–for example, questionnaires, surveys, and interviews–to collect personal opinions. Realizing their subjective limitations, recent studies have sought ways to leverage crowd-sourced data proliferating on the web to understand the subjective aspects of our cities through an objective viewpoint. However, to date textual data have not been favored for research purposes, as language barriers remain a significant obstacle. In this context, this research seeks to demonstrate the research potential of social media text data in the field of urban studies and develops a visualization that expresses collective perceptions referring to the concept of conventional cognitive maps.

The results of the study successfully answered the research questions. First, the overlay analysis between the distribution maps of check-in points and hashtag keywords revealed several discordant locations. This suggests that representative locations can be more accurately selected by taking textual data into consideration. We proceeded on the basis of the place identity components provided by Relph [4]: physical setting, activity, and meaning. Hashtags were assigned with class values that signify one of the place identity elements and were visualized in an intuitive image through a computational method, which we term a “crowd-sourced cognitive map”, to represent the collective perceptions of people. Place-specific features were pointed out to confirm the visualization’s effectiveness in representing place identity. Furthermore, the resulting images revealed that green spaces take precedence over other places in the overall urban identity formation process in newly developed cities.

When analyzing the results of this research, we found some limitations that should be considered in future work. First, a better-established definition of place identity and its components should be applied. This study adopted Relph’s model [4], whereas recent studies accept definitions that view place identity as a multidimensional concept. This subdivision in the latest debates becomes crucial in clarifying the semantics of the “meaning” element, which was distinguished in our analysis. Second, we scarcely reduced the problem of the language barrier, which was proposed as a major constraint in using text data, regardless of its effectiveness in delivering people’s perceptions [15]. Third, further developments are required to elaborate the visual representation. Denoting subjective contents–activity and meaning–with representative symbols or expressing spatial relations into a network can be possible alternatives to obtain an intuitive image of urban identity. A specific example is the self-organizing map (SOM) technique [40,41], which provides directions on how to relate textual attributes with each other based on their similarity in a visual format. Lastly, Instagram is often considered a biased media in terms of users’ age, income, or lifestyle. Indeed, the result of a survey on mobile social network service (SNS) usage rate with age shows that nearly 90 percent of Instagram users, particularly in Korea, range from the teens to the thirties [42]. Although we collected a large amount of data to objectify the place identity of a specific city, whether the developed method properly represents perceptions of all demographics remains to be clarified in further studies.

Despite the above shortcomings, our research approach produced various contributions for the quantification of place identity. By successfully translating textual data into place identity elements, we opened the possibility for the use of social media text data in capturing the identity of cities. Moreover, we suggested a graphical representation of a multicentric map through which people could apprehend the overall image of the city. Each set of circles in the plot contains information about multiple locations regardless of their spatial features, whether a node, street, or district. To urban designers, planners, and city officials, the work in this paper is expected to provide a methodological technique for appropriate decision-making and the evaluation of urban identity in order to help shape a more imageable city.
